# Dermatology

**Published:** 1876-11

**Authors:** 


DERMATOLOGY.—On account of the complicated character of diseases of the skin, and the increase of this complication by the obscurity of most treatise on this subject, almost every one in general practice suffers more or less difficulty in deciding upon a correct and satisfactory diagnosis and treatment. None but those devoting exclusive attention to dermatology can comprehend the multifold divisions of eczema, erethema, lichen, impetigo, etc., and are lost and confused by the extent and multiplicity of the technical terms used to present a comprehensive (?) pathology of skin diseases.

What the profession needs is some practical work on the subject—something that, dealing not exclusively in obscure terms and endless varieties, would present facts, plain and comprehensive facts. For this purpose the explanation by obscure terms must be abolished, and in their place substituted detailed accounts and comprehensive dissertations on the various pathological changes and etiological peculiarities met with in the leading classes of these diseases.

What is more calculated, for instance, to confuse the beginner, or the general practitioner even, than the almost endless division of varieties of erythema, into furgax, circinatum, intertrigo, læve, etc. This may be said of lichen, eczema, and all the rest of the primary divisions, and it might be readily seen what an amount of confusion it is bound to leave the mind in when ever we attempt to inform ourselves on the subject.

It is hoped that the American Dermatological Association, the organization of which is given below, will make, as one of its leading features, an attempt to simplify the nomenclature of dermatology. No fairer auspices could surround it, nor a better element of success and prosperity be desired. By it a practical, comprehensive treatise may be presented to the profession, and thereby relieve the learner in the study of these loathsome and troubsome affections that infest the land.

The American Dermatological Association was organized on Wednesday, September 6th, 1876, in accordance with the following call:

NEW YORK, July . . 1876.

DEAR SIR—At an informal meeting of the undersigned, held in Philadelphia, at the rooms of the Section of Practical Medicine, after the election of a Chairman and Secretary, *pro tem.*, it was *Resolved*, To call upon such American physicians as had evinced a special interest in dermatology, to unite in forming an American Dermatological Association. *Resolved*, That the meeting for organization be held in the University of Pennsylvania, Philadelphia, on Wednesday, September 6th, 1876, at 6 P.M., or immediately after the close of the meeting of the Section of Dermatology and Syphilology, of the International Medical Congress on that day. It is sincerely desired that you will be present and aid in the organization. Please signify your pleasure to the Secretary at the earliest opportunity, and oblige,

Very truly yours,

L. D. BULKLEY, Secretary, *pro tem.*,

1 East 33d St., New York.

LOUIS A. DUHRING,

Philadelphia, Pa.

LUNSFORD P. YANDELL, JR.,

Louisville, Ky.

GEORGE HENRY FOX,

New York.

EDW. WIGGLESWORTH,

Chairman,

Boston, Mass.

J. E. ATKINSON,

Baltimore, Md.

This call was sent to about fifty physicians in the various parts of the United States, whose names were known in connection with dermatology, and suggested by the informal committee issuing the call.

Twenty-three gentlemen responded, and these, together with the six signers of the call, comprise the twenty-nine original members of the Association.

The meeting for organization was called to order by the chairman of the committee issuing the call, Dr. Wigglesworth, of Boston, on Wednesday, September 6th, 1876, at six o'clock. The following gentlemen were present at the organization: Drs. J. E. Atkinson, Baltimore, Md.; D. R. Brown, Baltimore, Md.; L. Duncan Bulkley, New York; S. C. Busey, Washington,

D. C.; Louis A. Duhring, Philadelphia; C. Heitzman, New York; E. L. Keyes, New York; J. A. Octerlony, Louisville, Ky.; H. G. Piffard, New York; R. W. Taylor, New York; Arthur Van Harlingen, Philadelphia; F. D. Weisse, New York; Jas. C. White, Boston; Edward Wigglesworth, Jr., Boston.

Dr. Wigglesworth was continued in the chair, and Dr. Bulkley elected secretary of the meeting for organization, and committee was appointed, consisting of Drs. Bulkley, Octerlony, and Van Harlingen, to prepare a constitution and by-laws and to make nominations for officers.

The constitution and by-laws offered were discussed, amended and adopted, section by section, and at an adjourned meeting on Thursday, September 7th, they were adopted *in toto*, the American Dermatological Association was declared organized, and the following officers were elected for the coming year:

President—James C. White, M.D., of Boston.

Vice-Presidents—Louis A. Duhring, M.D., of Philadelphia; R. W. Taylor, M.D., of New York.

Secretary—L. Duncan Bulkley, M.D., of New York.

Treasurer—James Nevins Hyde, M.D. of Chicago.

The Association then adjourned to meet on the first Tuesday in September, 1877, at Niagara Falls.—*Arch. Der.*

